# Impacts of *Metarhizium brunneum* F52 infection on the flight performance of Asian longhorned beetles, *Anoplophora glabripennis*

**DOI:** 10.1371/journal.pone.0221997

**Published:** 2019-09-06

**Authors:** Eric H. Clifton, Jason Cortell, Linqi Ye, Thomas Rachman, Ann E. Hajek

**Affiliations:** 1 Department of Entomology, Cornell University, Ithaca, New York, United States of America; 2 Dynamic Locomotion, Inc., Groton, New York, United States of America; 3 Center for Artificial Intelligence and Robotics, Graduate School at Shenzhen, Tsinghua University, Shenzhen, China; Montana State University Bozeman, UNITED STATES

## Abstract

The Asian longhorned beetle (ALB), *Anoplophora glabripennis*, is an invasive wood-borer in North America and Europe that threatens a variety of tree genera, including *Acer* and *Populus*. All invasive ALB populations occur in quarantine zones where they are under eradication, a process that is difficult and expensive, requiring extensive surveys and host tree removals. Although ALB has been described as an insect that is typically slow to disperse, some rare individuals that fly longer distances have the potential to start infestations outside of quarantine zones. Biological control using entomopathogenic fungi has been considered as another option for managing ALB infestations. The entomopathogenic fungus *Metarhizium brunneum* strain F52, registered for commercial use in the United States, is effective at killing ALB adults but information is lacking on how this entomopathogen affects ALB flight behavior before death. Using quarantine-reared ALB, flight mills were used to collect data on flight performance of beetles at multiple time points after infection. Healthy (uninfected) male ALB adults always flew significantly greater distances than females. The maximum observation for total flight distance was a healthy male that flew 10.9 km in 24 hours on a flight mill. ALB adults infected with *M*. *brunneum* F52 flew significantly shorter distances compared to healthy adults, starting one week after fungal exposure. Biological control of ALB with this fungal entomopathogen could help to reduce their dispersal in the environment and, thereby, decrease the risk of adults moving outside of quarantine zones.

## Introduction

The Asian longhorned beetle (ALB), *Anoplophora glabripennis*, is an invasive, cryptic woodborer in North America and Europe that threatens many tree genera, including *Acer* and *Populus*, that are abundant in forests and populated areas [1,2]. Since the first discovery of ALB in North America in 1996, all ALB populations have been subjected to quarantine and eradication efforts. Within quarantine zones, intensive surveys are conducted to identify infested trees (typically through the detection of oviposition pits left by adult females) and to destroy infested host material. In some cases, prophylactic applications of systemic insecticides are used on host trees in areas of high risk [3]. ALB infestations have been found in 5 eastern states in the USA. Successful eradication has been achieved in Illinois and New Jersey, and for some populations in New York and Massachusetts, using these methods [2]. As of May 2019, ongoing ALB eradication programs continue in areas of Massachusetts, Ohio, and New York [4].

The sizes of areas that are quarantined around ALB-infested trees are partially based on the known biology and dispersal behavior of ALB. Field studies using mark-recapture or harmonic radar in China have suggested that ALB is not a frequent flyer and often does not disperse more than 2 km in a lifetime [5–7]. However, these studies noted that some beetles escaped detection or recapture, and those individuals could disperse greater distances that are impossible to measure using these methods. Studying the dispersal behavior of ALB populations in non-native habitats presents challenges, considering the differences in landscapes and host trees, and the eradication efforts that are in place to quickly remove infested trees. Dispersal models based on field data from ALB-infested trees in the Worcester, MA quarantine suggested that longer distance dispersal events could rarely be occurring in the field, with the result that these beetles could escape from quarantine zones [8]. In agreement, recent studies using computerized flight mills to measure ALB flight performance found that 5% of individuals were capable of flying >8 km during 24 h trials or the sum of repeated 4 h trials, and a few adults flew >13 km [9,10]. Keena [11] studied the flight propensity of ALB adults and provided a good review of environmental factors and population dynamics that could affect their flight behavior in the field.

While ALB eradication programs in North America and Europe are primarily focused on removals of infested trees, use of entomopathogenic fungal biopesticides could add another option toward ALB eradication [1,12]. Previous studies have determined that fungal entomopathogens, including *Metarhizium* spp. and *Beauveria* spp., can infect and kill ALB [13,14]. In particular, *Metarhizium brunneum* (formerly *M*. *anisopliae* [15]) strain F52 has shown promise as a biological control agent for ALB [16–21]. Lethal, infective spores of *M*. *brunneum* F52 can be transmitted horizontally between adults during mating and oviposition and infection can reduce ALB oviposition [22,23]. Ideally, *M*. *brunneum* F52 could be used in a prophylactic manner on high-risk, susceptible trees in a quarantine zone to infect ALB adults that emerge before tree removal or those that emerge from ALB-infested trees that have escaped detection.

A few studies have assessed how pathogens and parasites can negatively affect the flight behavior of insects, including European corn borer (*Ostrinia nubilalis*) infected with microsporidia (*Nosema pyrausta*) [24], honeybees (*Apis mellifera*) infected with microsporidia (*Nosema ceranae*) or deformed wing virus (DWV) [25], and monarch butterflies (*Danaus plexippus*) infected with an apicomplexan parasite (*Ophryocystis elektroscirrha*) [26]. Honey bees infected with DWV flew mean distances of 150 m compared to healthy honeybees that flew 480 m [25]. Parasitized monarch butterflies flew 19% shorter distances than healthy individuals [26]. All of these studies were conducted on parasites that are more chronic and are not known for rapid lethality in the field. To our knowledge, only two published studies have tested whether pathogens and parasites affect the flight behavior of wood-boring beetles, and both of these studies involved parasitic nematodes. Akbulut and Linit [27] found that *Monochamus carolinensis* flight distance and duration was significantly shortened after inoculation with larger concentrations of the pinewood nematode (*Bursaphelenchus xylophilus*). Forsse [28] noted that parasitic nematodes had no impact on the flight performance of the bark beetle *Ips typographus*.

The primary goal for using a lethal biological control agent like *M*. *brunneum* F52 is to kill ALB adults before oviposition would occur. However, information is lacking on how infection by this fungus could affect ALB flight behavior and whether infection influences the capacity of ALB to disperse. During the later stages of infection and before *M*. *brunneum* F52 kills its host, infected ALB adults feed less and exhibit visible lethargy compared to healthy adults. While laboratory studies with flight mills may exaggerate the flight capacity of insects [10,29], they are great substitutes for dispersal experiments that cannot be easily performed in the field and for comparing insects subjected to different experimental treatments. In the current study, we report on how the lethal fungal entomopathogen *M*. *brunneum* F52 affects ALB flight performance and how this could benefit eradication efforts.

## Materials and methods

### Fungal isolate

*Metarhizium brunneum* strain F52 (ARSEF 7711) was obtained from non-formulated conidial powder (infective spores) produced with solid state fermentation by the USDA-ARS in Sidney, Montana. One to two days before bioassays, the conidial viability was determined by spreading a conidial suspension on Sabouraud dextrose agar [30]. Germinated conidia were counted under optical magnification (400x) 14–18 h after incubation at 25°C.

### Test insects and bioassay procedures

Asian longhorned beetles were reared, inoculated and flown under USDA-APHIS permit (# P526P-17-01304) in the Sarkaria Arthropod Research Laboratory (SARL), a quarantine lab at Cornell University. Rearing followed methods described in the Supplementary Appendix of Goble et al. [16]. Prior to and during bioassays, after beetles had been removed from flight mills, beetles were held in individual 473 mL clear plastic containers at 25°C:15°C day:night with 14:10 h L:D. Striped maple (*Acer pensylvanicum*) twigs with leaves removed were used for feeding. Beetles had eclosed (emerged from pupal cases and sclerotized) 10 to 13 days (mean ± SE: 12.0 ± 0.1 days) prior to beginning bioassays. Only unmated beetles were used in this study.

We inoculated adult beetles with conidia using methods developed by Dubois et al. [13], with slight modifications. For a consistent exposure concentration, beetles were submerged in individual 50 mL polystyrene tubes containing 11–13 mL of suspensions of 10^7^ viable conidia mL^-1^ in 0.05% sterile Tween® 80 surfactant (Acros Organics, The Hague, Netherlands; CAS 9005-65-6), and shaken gently by hand for 5 seconds. This treatment typically results in median survival times of 16 days for males and 14 days for females, based on preliminary experiments with the F52 strain (EHC, unpublished data). We used a dose of 10^7^ conidia mL^-1^ in the bioassays because higher doses with this lethal pathogen will kill beetles quickly and before there would be time to perform flight mill trials at multiple time points during infection (S1 Fig). The same inoculation method was used for the control treatment (0.05% sterile Tween® 80 surfactant).

### Experimental design

**Fig 1:**
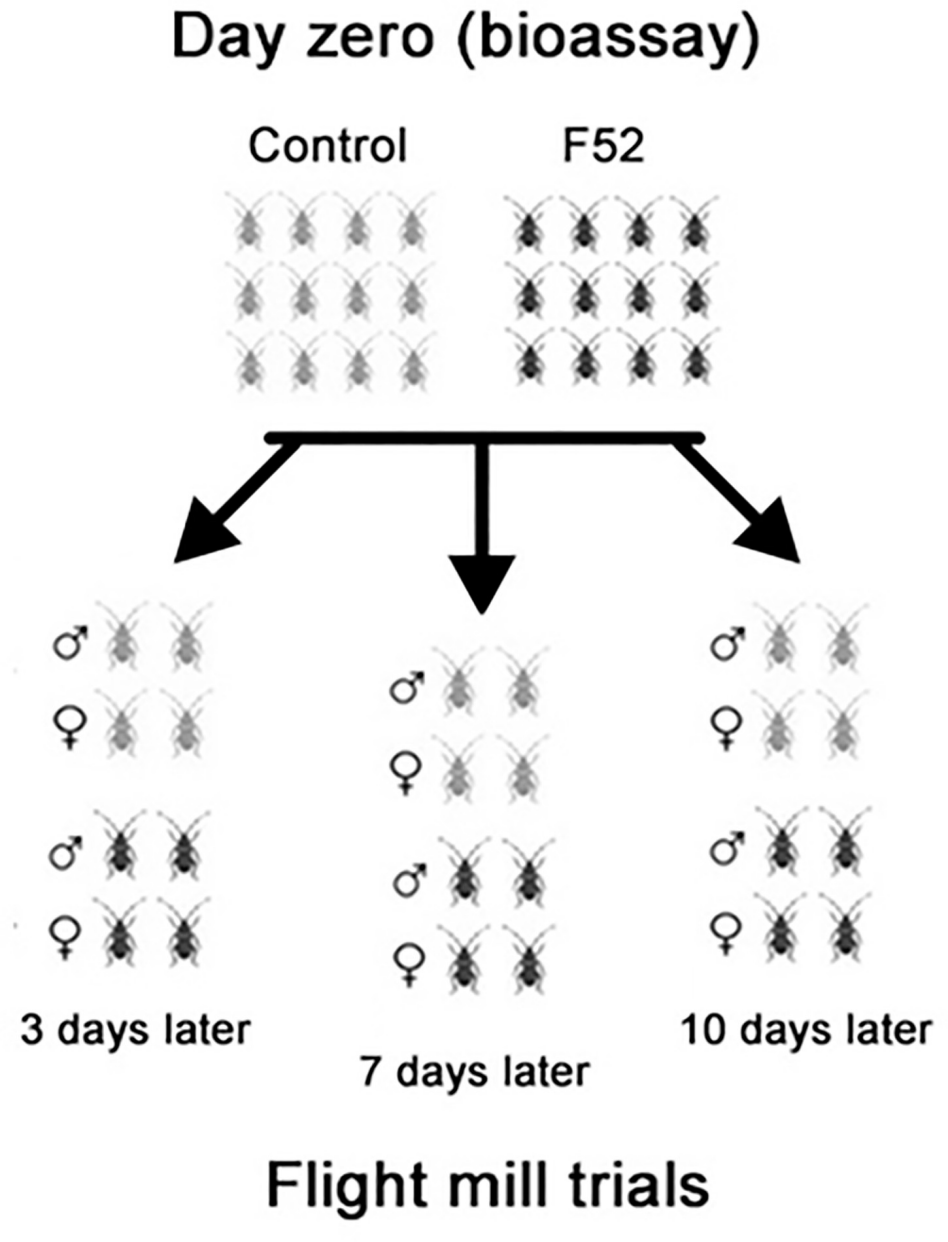
Experimental design. Schematic representing the bioassays and the time points for flight mill trials.

We used computerized flight mills in the quarantine lab to compare the flight performance of healthy ALB adults to those infected with *M*. *brunneum* F52 (hereafter referred to simply as F52). On dates of experiment setup (day zero), beetles (1:1 ratio of male:female) were exposed to suspensions of F52 conidia or sterile 0.05% Tween®, as described above. Bioassays and flight trials were conducted between June 2018 and January 2019. These experiments had to be conducted using beetles from 16 bioassays due to limited space that could house only eight large flight mill arenas (S1 Table). The experimental design was fully crossed with 2 sexes (male and female) × 2 treatments (control and F52) × 3 time points [3 days after treatment (DAT), 7 DAT, 10 DAT]. At each of these time points, 2 male and 2 female ALB adults were randomly selected from both F52-treated and control beetle groups and were assigned to flight mill trials for 24 hours (Fig 1). Therefore, each 24 h flight included both sexes and both treatments and beetles from only one DAT group, resulting in a total of 38 flight mill trials (9 trials for 3 DAT, 15 for 7 DAT, and 14 for 10 DAT; S1 Table). Each beetle was flown only once (no repeated measures). Beetles were not used for flight trials on day zero, the same day as bioassays, because F52 conidia could be inadvertently removed from beetle cuticles during handling and attachment to flight mills, resulting in less consistent exposures. Based on the dose of F52 we used for bioassays, we included the 3 DAT time point to verify that there is a time lag between fungal infection and measurable declines in flight performance. After acquiring sufficient data to show that there were no significant differences at 3 DAT (*n* ≥16 beetles per treatment for either sex, exposed during 9 different bioassays), we discontinued flight mill experiments for this time point and focused further trials on the later time points of 7 DAT and 10 DAT (*n* = 27–30 beetles per treatment for either sex). Before and after flight mill trials, beetles were checked daily for mortality and experiments ended 28 DAT.

### Recording flight parameters with computerized flight mills

Flight mill trials were conducted inside a walk-in environmental chamber (10.2 m^2^, 23°C, 63% RH). The chamber ceiling was lit with eight 32W fluorescent T8 bulbs. The photoperiod inside the walk-in chamber was 14:10 h L:D, starting at 06:00 h and ending at 20:00 h. On a day of flight mill trials, flights began between 09:00 h and 12:00 h. Flight mills were kept inside lidded, circular arenas on a level table in the chamber to prevent beetle escape in case they accidentally detached from flight mills. Arena walls were constructed with opaque plexiglass acrylic sheets (thickness = 1.60 mm) and secured together with duct tape. Each circular arena had a diameter of 46 cm and stood 25.4 cm tall. Panels of clear plexiglass acrylic (thickness = 5.50 mm) were placed on top of arenas as lids.

**Fig 2:**
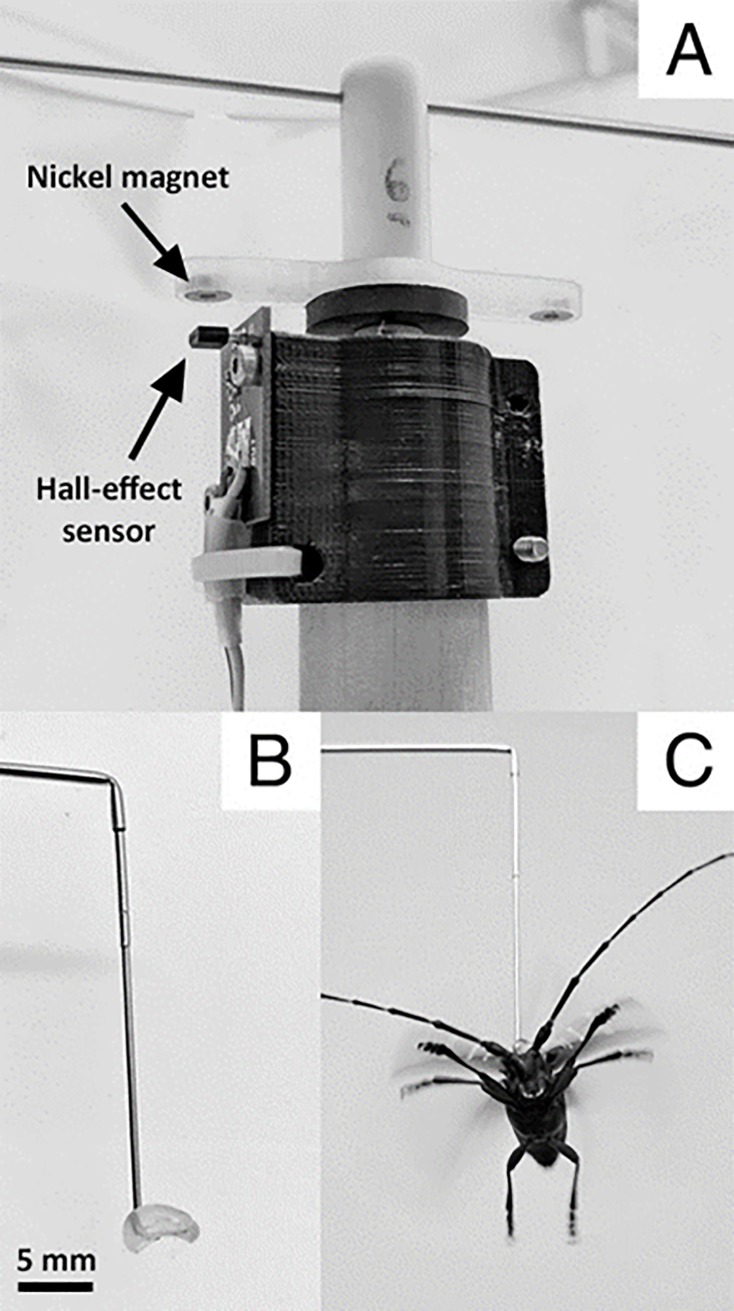
Flight mills and beetle attachment. (A) Hall-effect sensor attached to flight mill that detects rotations of the magnets, located on opposite ends of the Teflon bearing that holds the flight mill arm; (B) insect pin inserted into flight mill arm; a bead of adhesive is applied to the L-shaped end of the insect pin to tether a beetle by the pronotum; (C) tethered *Anoplophora glabripennis* adult attached to flight mill arm.

Flight mills were custom-built and based on the design used by Jones et al. [31], with slight modifications. For the flight mill arms (FMA), we used miniature stainless steel hypodermic tubing (internal diameter = 0.61 mm) with lengths of 31.8 cm and one end bent 90^o^. An ALB adult was attached to one end of the flight mill arm (described in more detail below). As the ALB adult flew in circles (1 m circumference), small nickel magnets below a Teflon bearing were detected by a digital Hall-effect sensor (Fig 2A), to record rotations of the FMA. The wires for the Hall-effect sensors were attached to a data acquisition device (model USB-6008, National Instruments, Austin, TX) connected to a laptop computer. Raw data were collected with customized LabVIEW software (National Instruments, Austin, TX), modified from Jones et al. [31]. The software provided a summary of the total distance flown, flight velocity and the number of flight bouts (movement of more than 5 s before a complete stop). Raw data were also summarized using Visual Basic for Applications (VBA) macros in Microsoft Excel (Microsoft Corporation, Washington, U.S.), which calculated the times and distances for each flight bout, as well as the total flight duration for each 24 h trial (S1 File).

### Attachment of beetles to flight mills

Prior to attachment to flight mills, beetles were weighed with a precision balance (model XS4002S, Mettler-Toledo, Columbus, OH) and elytron length was measured with digital calipers (VWR, Cat No. 62379–531). Beetles were tethered by the pronotum to a #5 insect pin (0.60 mm thickness). Before tethering beetles, the cap on the dull end of the insect pin was removed and the terminal 2–3 mm was bent at a 90° angle using pliers to form an L-shape (Fig 2B). Beetles were attached to this short section with hot melt adhesive (AdTech, multi-temp glue sticks; Hotmelt, Edina, Minnesota, USA), applied with a glue gun (AdTech 2Temp®, model 0453) on the low-temperature setting (149°C) (Fig 2C). Using pliers, the insect pin with the tethered beetle was carefully inserted into the vertical, bent end of the FMA, so that beetles were then facing perpendicularly (90°) to the length of the FMA. Similar to Lopez et al. [9], we used a piece of modeling clay at the opposite end of the FMA to counterbalance the weight of each beetle and confirmed that individual beetles could open and move their wings freely. Beetles were not stimulated to fly, but most beetles (>90%) initiated flight as soon as they were placed inside the flight mill arenas after being tethered and then attached to the FMA. Javal et al. [10] presented ALB adults with plastic pipes to mimic a twig and stimulate flight, but their trials were shorter in duration. In the present study, after the 24 h flights, beetles were carefully detached from flight mills and tethering pins, returned to their original container, and checked daily for mortality.

In total, 292 beetles were attached to flight mills for 24 hr trials (146 inoculated with F52; 146 controls). Lopez et al. [9] excluded ALB that did not fly from their analyses; however, assuming that F52 infection could affect flight performance, we included beetles that did not fly, although this was rare (<1% of controls and 5% of F52-treated beetles).

### Data analysis

We used mixed-model analysis of variance (ANOVA), with the PROC MIXED statement in SAS Enterprise 7.15 [32]. We analyzed data separately by sex because males flew significantly more than females across all time points. Treatments (control vs. F52), time points (3, 7, or 10 DAT), and their interaction were used as fixed effects in the models. We included the pre-flight weight (g) and elytron length (mm) of each beetle as quantitative variables. Bioassay replicate was included as a random effect in all of the analyses. We analyzed data for total flight distance, total flight duration, average flight velocity, the number of flight bouts, the average flight duration per bout, and the average distance per bout. There was no need to transform data because residuals had normal distributions. Pairwise comparisons for treatment (control vs. F52) were made at different time points using the PDIFF statement in PROC MIXED. Pairwise comparisons were based on least-square means with an experiment-wise significance level of *P* < 0.05 based on a Tukey-Kramer adjustment for multiple comparisons. We also compared the survival times of males and females treated with F52 using PROC MIXED, using sex as the fixed effect and combining data for beetles flown during all time points. Assuming that F52 infection would kill beetles at a faster rate if they expended more energy, we also performed an analysis of covariance (PROC GLM) [32] on the survival times of the F52-treated beetles using total flight distance as a covariate and combining data for all time points.

## Results

No control beetles died within 28 DAT. For beetles treated with F52, 92% of females and 89% of males died within 28 DAT. Females treated with F52 had significantly shorter survival times (mean: 14.6 ± 0.5 d) than males treated with F52 (mean = 18.0 ± 0.5 d) (df = 115; t = 2.48; *P* = 0.0147).

According to the analysis of covariance for F52-treated beetles, there was no significant effect of total flight distance on the survival times of females. In contrast, there was a significant effect of flight distance on the survival time of males treated with F52 (S2 Fig); however, this only explained ~9% of the variation and may simply reflect how the males with longer survival times had overall greater flight performance at the time of flight mill trials.

**Fig 3:**
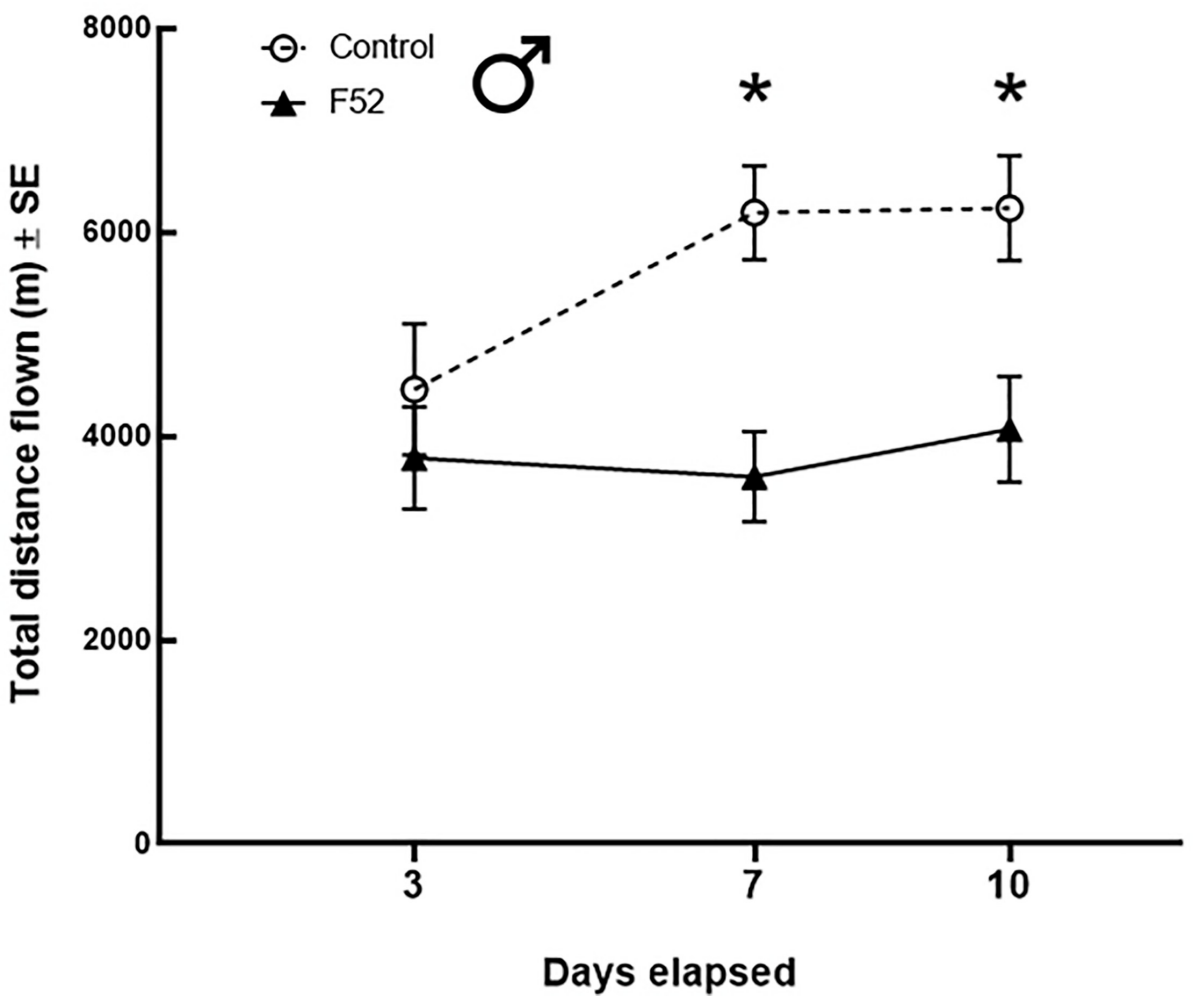
Male beetle flight performance. Average (mean ± SE) total distance flown (m) for *A*. *glabripennis* adult males under different treatments and time points after exposure. Asterisks denote a significant difference between treatments (F52 vs. control) at a particular time point.

**Fig 4:**
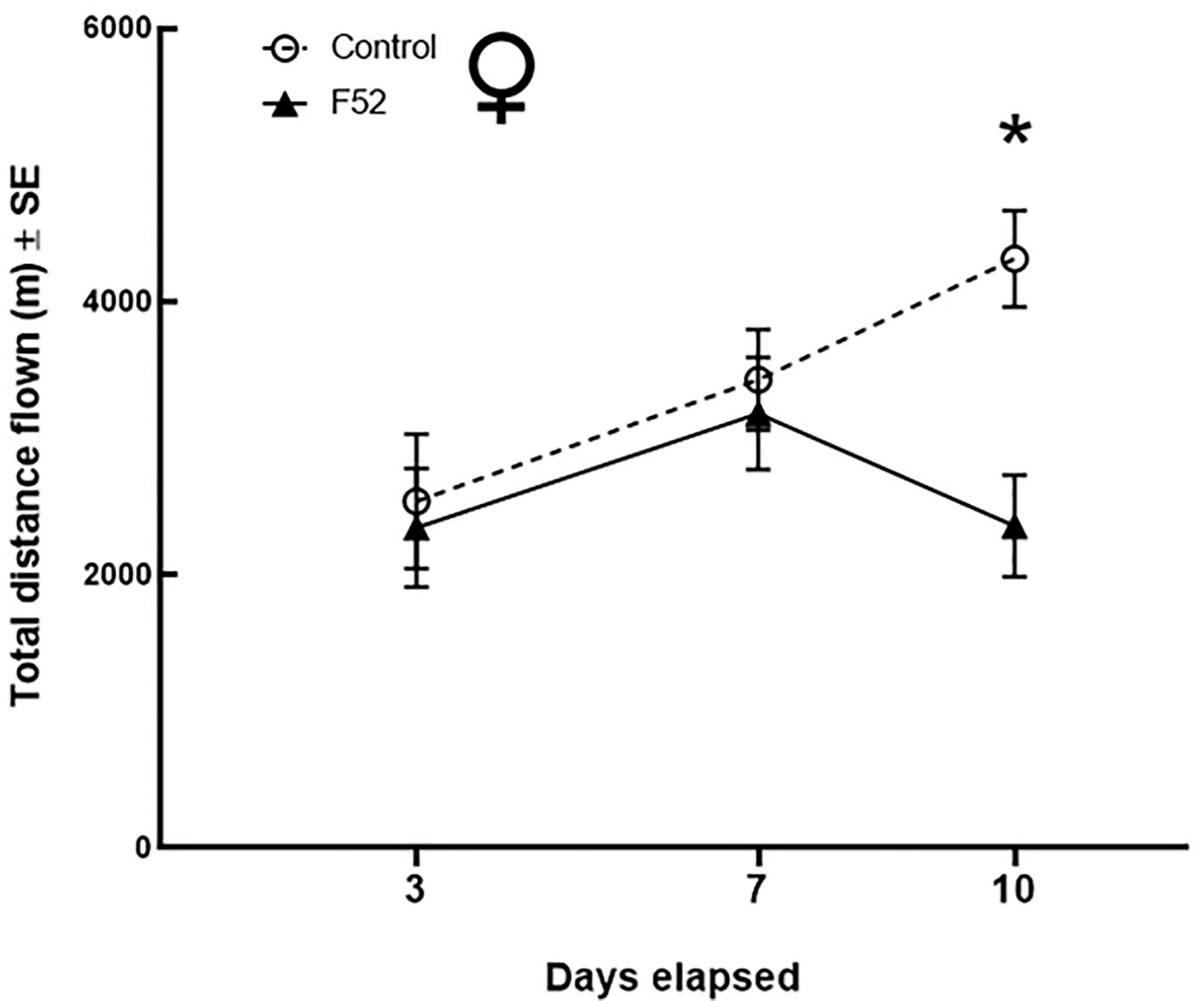
Female beetle flight performance. Average (mean ± SE) total distance flown (m) for *A*. *glabripennis* adult females under different treatments and time points after exposure. Asterisks denote a significant difference between treatments (F52 vs. control) at a particular time point.

**Fig 5:**
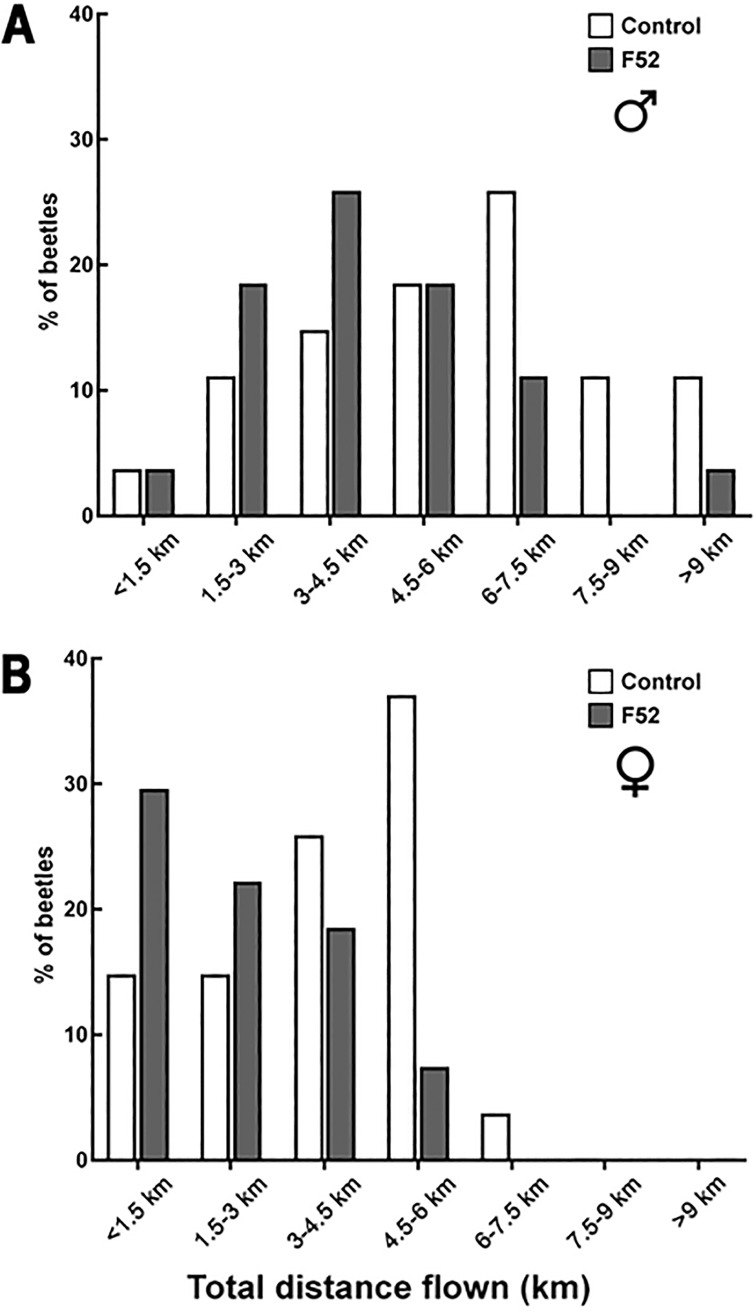
Distribution of total distances at 10 days after treatment. Percentage of healthy and F52-treated *A*. *glabripennis* (A) males and (B) females sorted by total distance (km) flown during 24-hour trials performed 10 days after treatment. Compared to the F52 treatment, we observed a higher percentage of beetles in the control treatment that flew longer distances.

The maximum observation for total distance was from a male beetle in the control treatment that flew 10,941 m during the 24-h flight trials. 14% of males (*n* = 20) flew >8 km during 24-h flight trials. None of the females flew >8 km, but 10% (*n* = 15) flew >6 km during 24-h flight trials. At 3 DAT, there were no significant differences between treatments for the total distance flown by males and females (Table 1). At 7 DAT, F52 significantly reduced the total distance flown for males by ~42% (df = 125; t = 3.71; *P* = 0.0041; Fig 3; Table 2), but not for females (Fig 4). At 10 DAT, F52 significantly reduced the total distance flown by ~35% for males (df = 125; t = 3.23; *P* = 0.0194) and ~45% for females (df = 121; t = 3.69; *P* = 0.0045; Figs 3 and 4; Table 3). Additionally, at 10 DAT, there was a higher percentage of healthy (control) beetles that flew >4.5 km compared to F52-treated beetles (Fig 5). For both females (Table 4) and males (Table 5), body weight and elytron length had no significant effects on total flight distance.

**Table 1:** Average (mean ± SE) flight parameters for *A*. *glabripennis* adults under varying sex and treatment, 3 days after treatment. Means followed by different lowercase letters are significantly different between treatments within sex.

	3 days after treatment			
	Male		Female	
Flight Parameter	Control	F52	Control	F52
Total distance flown (m)	4457.64 ± 643.30 a	3785.47 ± 550.39 a	2537.79 ± 491.01 a	2345.37 ± 432.67 a
Total flight time (min)	37.87 ± 5.63 a	30.08 ± 3.61 a	24.72 ± 4.75 a	21.78 ± 4.56 a
Flight velocity (m/s)	1.94 ± 0.06 a	2.02 ± 0.08 a	1.70 ± 0.10 a	1.72 ± 0.16 a
Number of flight bouts	6.35 ± 0.91 a	7.35 ± 0.99 a	8.76 ± 1.88 a	8.12 ± 3.89 a
Flight bout time (s)	432.08 ± 68.76 a	322.58 ± 61.07 a	203.17 ± 37.88 a	283.26 ± 73.36 a
Distance per bout (m)	848.37 ± 130.15 a	672.70 ± 130.87 a	359.24 ± 67.79 a	518.11 ± 108.77 a
Elytron length (mm)	17.11 ± 0.14	17.43 ± 0.24	19.67 ± 0.24	19.64 ± 0.21
Pre-flight weight (g)	0.77 ± 0.02	0.88 ± 0.04	1.08 ± 0.04	1.09 ± 0.04
*n*	17	17	17	16

**Table 2:** Average (mean ± SE) flight parameters for *A*. *glabripennis* adults under varying sex and treatment, 7 days after treatment. Means followed by different lowercase letters are significantly different between treatments within sex.

	7 days after treatment			
	Male		Female	
Flight Parameter	Control	F52	Control	F52
Total distance flown (m)	6191.60 ± 459.30 a	3600.34 ± 443.03 b	3427.76 ± 368.64 a	3180.02 ± 411.93 a
Total flight time (min)	51.57 ± 3.85 a	30.18 ± 3.56 a	30.40 ± 3.18 a	28.03 ± 3.66 a
Flight velocity (m/s)	2.02 ± 0.05 a	1.90 ± 0.08 a	1.81 ± 0.08 a	2.06 ± 0.28 a
Number of flight bouts	9.90 ± 1.29 a	5.63 ± 0.65 b	6.36 ± 1.04 a	6.00 ± 1.04 a
Flight bout time (s)	382.69 ± 27.71 a	385.04 ± 45.61 a	345.79 ± 37.08 a	363.50 ± 43.59 a
Distance per bout (m)	775.97 ± 58.64 a	755.30 ± 85.04 a	652.13 ± 70.38 a	764.42 ± 95.16 a
Elytron length (mm)	17.68 ± 0.12	17.32 ± 0.12	19.91 ± 0.15	19.42 ± 0.15
Pre-flight weight (g)	0.88 ± 0.02	0.80 ± 0.02	1.15 ± 0.03	1.03 ± 0.03
*n*	30	30	28	29

**Table 3:** Average (mean ± SE) flight parameters for *A*. *glabripennis* adults under varying sex and treatments, 10 days after treatments. Means followed by different lowercase letters are significantly different between treatments within sex.

	10 days after treatment			
	Male		Female	
Flight Parameter	Control	F52	Control	F52
Total distance flown (m)	6236.02 ± 514.08 a	4066.77 ± 518.56 b	4314.81 ± 353.55 a	2359.13 ± 372.54 b
Total flight time (min)	51.99 ± 4.10 a	34.53 ± 4.35 b	37.23 ± 3.39 a	22.29 ± 3.31 b
Flight velocity (m/s)	1.92 ± 0.08 a	1.68 ± 0.15 a	1.96 ± 0.07 a	1.53 ± 0.15 a
Number of flight bouts	8.04 ± 1.10 a	7.08 ± 0.95 a	6.44 ± 0.71 a	10.70 ± 4.51 a
Flight bout time (s)	479.26 ± 44.46 a	346.97 ± 44.16 a	384.96 ± 33.07 a	247.42 ± 34.37 a
Distance per bout (m)	958.81 ± 89.70 a	714.99 ± 100.03 a	770.86 ± 71.79 a	480.90 ± 73.50 a
Elytron length (mm)	17.33 ± 0.19	17.43 ± 0.16	19.62 ± 0.18	19.60 ± 0.15
Pre-flight weight (g)	0.82 ± 0.03	0.82 ± 0.03	1.06 ± 0.03	1.08 ± 0.03
*n*	27	27	27	27

**Table 4:** Analysis of variance for flight data of female *A*. *glabripennis* adults.

Analysis	Effect	df	F	P
Total flight distance	Weight	1, 121	0.35	0.5540
	Elytron length	1, 121	0.16	0.6875
	Treatment^a^	1, 121	6.79	0.0103*
	Time point^b^	2, 121	2.29	0.1052
	Treatment × time point	2, 121	2.71	0.0708
Total flight duration	Weight	1, 121	0.08	0.7763
	Elytron length	1, 121	0.23	0.6326
	Treatment	1, 121	5.48	0.0208*
	Time point	2, 121	1.28	0.2806
	Treatment × time point	2, 121	1.63	0.2007
Average velocity	Weight	1, 121	0.35	0.5564
	Elytron length	1, 121	1.32	0.2526
	Treatment	1, 121	0.22	0.6389
	Time point	2, 121	1.00	0.3706
	Treatment × time point	2, 121	2.22	0.1135
Number of flight bouts	Weight	1, 121	0.18	0.6704
	Elytron length	1, 121	0.85	0.3582
	Treatment	1, 121	0.17	0.6836
	Time point	2, 121	0.63	0.5362
	Treatment × time point	2, 121	0.62	0.5372
Average bout duration	Weight	1, 121	0.26	0.6085
	Elytron length	1, 121	1.02	0.3142
	Treatment	1, 121	0.07	0.7926
	Time point	2, 121	3.05	0.0509
	Treatment × time point	2, 121	3.33	0.0390*
Average distance per bout	Weight	1, 121	0.16	0.6874
	Elytron length	1, 121	0.39	0.5326
	Treatment	1, 121	<0.01	0.9694
	Time point	2, 121	4.71	0.0108*
	Treatment × time point	2, 121	4.37	0.0148*

Asterisks denote significant effects in the model (*P*-value column).

^a^ Control suspension (0.05% sterile Tween® surfactant) or *Metarhizium brunneum* F52 (10^7^ conidia mL^-1^)

^b^ Days after treatment (3 DAT, 7 DAT, or 10 DAT)

**Table 5:** Analysis of variance for flight data of male *A*. *glabripennis* adults.

Analysis	Effect	df	F	P
Total flight distance	Weight	1, 125	0.77	0.3818
	Elytron length	1, 125	<0.01	0.9507
	Treatment^a^	1, 125	19.16	<0.0001*
	Time point^b^	2, 125	1.19	0.3074
	Treatment × time point	2, 125	0.99	0.3762
Total flight duration	Weight	1, 125	1.51	0.2217
	Elytron length	1, 125	0.26	0.6136
	Treatment	1, 125	21.45	<0.0001*
	Time point	2, 125	1.47	0.2330
	Treatment × time point	2, 125	0.59	0.5546
Average velocity	Weight	1, 125	0.35	0.5545
	Elytron length	1, 125	0.48	0.4877
	Treatment	1, 125	1.41	0.2367
	Time point	2, 125	2.03	0.1356
	Treatment × time point	2, 125	0.99	0.3747
Number of flight bouts	Weight	1, 125	1.67	0.1986
	Elytron length	1, 125	0.57	0.4511
	Treatment	1, 125	2.95	0.0884
	Time point	2, 125	0.14	0.8704
	Treatment × time point	2, 125	2.19	0.1168
Average bout duration	Weight	1, 125	0.08	0.7794
	Elytron length	1, 125	0.02	0.8876
	Treatment	1, 125	4.13	0.0442*
	Time point	2, 125	0.29	0.7498
	Treatment × time point	2, 125	1.19	0.3062
Average distance per bout	Weight	1, 125	0.09	0.7658
	Elytron length	1, 125	0.06	0.8088
	Treatment	1, 125	3.32	0.0707
	Time point	2, 125	0.38	0.6824
	Treatment × time point	2, 125	0.76	0.4696

Asterisks denote significant effects in the model (*P*-value column).

^a^ Control suspension (0.05% sterile Tween® surfactant) or *Metarhizium brunneum* F52 (10^7^ conidia mL^-1^)

^b^ Days after treatment (3 DAT, 7 DAT, or 10 DAT)

At 3 DAT, there were no significant differences between control and F52 treatments for the total flight duration by males and females (Table 1). At 7 DAT, F52 significantly reduced the total flight duration for males by ~39% (df = 125; t = 3.76; *P* = 0.0035; Table 2), but not for females (df = 121; t = 0.76; *P* = 0.9737). At 10 DAT, F52 significantly reduced the total flight duration by ~33% for males (df = 125; t = 3.15; *P* = 0.0242) and ~40% for females (df = 121; t = 3.02; *P* = 0.0352; Table 3). As with total flight distance, there was no effect of body weight or elytron length on total flight duration for females (Table 4) and males (Table 5).

Body size also had no significant effect on the flight velocity of ALB adults (Tables 4 and 5). For both males and females at 10 DAT, there was a numerical difference between treatments for flight velocities, but this difference was not significant (Table 3). Otherwise, there were no significant differences among flight velocities for all comparisons of treatment, time points, and their interaction (Tables 1–3).

**Fig 6:**
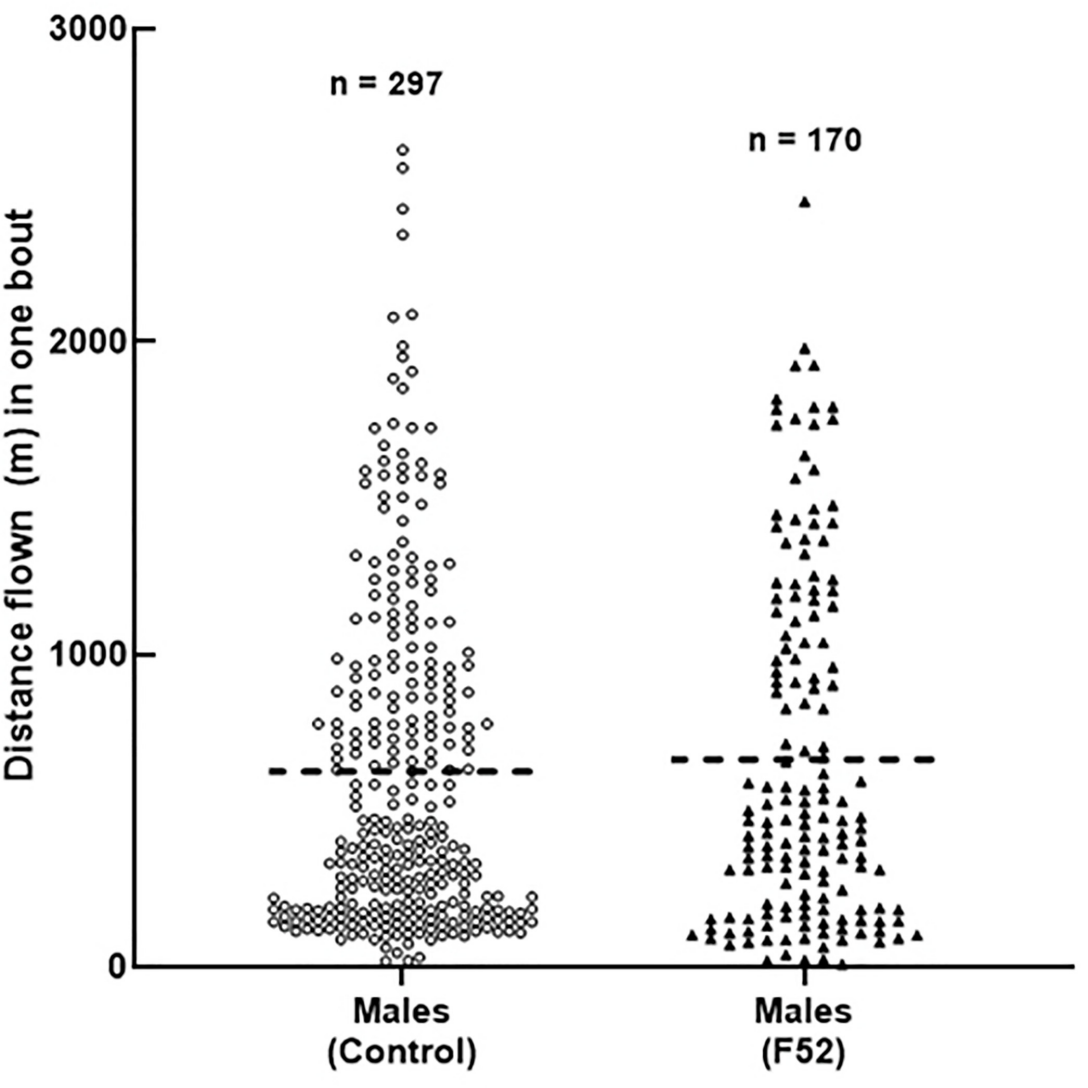
Male beetle flight bouts at 7 days after treatment. The distance flown (m) during individual flight bouts for *A*. *glabripennis* adult males, 7 days after treatment. Dotted line represents the mean. Sample size for total flight bouts is displayed above scatter plots.

At 7 DAT, males treated with F52 had significantly fewer flight bouts than controls, with almost half as many bouts (Table 2). The average distances per flight bout for males at 7 DAT were similar between treatments (Fig 6). However, the lower number of flight bouts for F52-treated males at 7 DAT accounts for the observed differences in total flight distance (Fig 3; Table 2). There were no significant differences between treatments for the number of flight bouts at 3 DAT and 10 DAT (Tables 1 and 3).

For both males and females, and for all DAT, there were no significant differences between treatments for the average durations of flight bouts and for the average distances of flight bouts (Tables 1–3). Although the differences for the average distance per bout were not significant at 10 DAT, there was a notable differences in the average values, with healthy individuals flying greater distances than F52-treated beetles at this time point (Table 3).

## Discussion

The present study demonstrates that F52 infection affected flight performance of males at 7 DAT and 10 DAT, and female flight performance was reduced at 10 DAT, based on the total distances flown during the 24-hr trials. This result is important because females initiate new infestations when they disperse and oviposit on new host trees. If they are infected with F52, toward the end of the infection, they do not fly as far. Although males do not start new infestations, they are still needed for successful reproduction, and infection also decreased the distance they flew later in infection. Even if these F52-infected males dispersed to different host trees in the environment, they could potentially spread infective conidia to females during mating events (horizontal transmission) [23]. At 10 DAT, there was a measurable difference between treatments for the average flight distance per bout (Table 3). Although this difference was not significant, this result suggests that the infected beetles would fly shorter distances than healthy beetles during individual flight events. Future studies with repeated measures could test the flight propensity of infected ALB adults and whether repeated forced-flight events and subsequent fatigue would affect the flight behavior of ALB and the lethality of *M*. *brunneum* F52.

We specifically chose the F52 dose of 10^7^ conidia mL^-1^ in the bioassays so that beetles did not die too quickly, thereby allowing for flight mill trials at multiple time points while infections progressed. Previous bioassays using an F52 dose of 10^8^ conidia mL^-1^ resulted in mean survival times of 12.2 ± 0.6 d for males and 10.3 ± 0.5 d for females (S1 Fig). Gardescu et al. [20] exposed ALB adults to F52 formulations on coated wood pieces, resulting in median survival times of ~9.5 d. Increasing F52 doses correlated with decreasing survival times for ALB adults [21], and one could assume that more rapid mortality than reported in this study would correspond with reduced flight performance at earlier time points after exposure.

Lopez et al. [9] used flight mills to measure flight parameters of ALB adults of varying age, mating status, and nutritional status (fed vs. starved). The same study found that nutrition and age had the greatest overall influence on flight capacity, with older (>5 d post-emergence from logs) and well-fed adults exhibiting significantly greater flight distances, durations, and numbers of flight bouts. Lopez et al. [9] used field populations of ALB that emerged from logs, whereas the adults from our quarantine colony completed eclosion in cups of artificial diet. Because it takes several days for eclosed adults to emerge from logs, we would assume that the age of the beetles we used (~12 d post-eclosion) resembles the age of “old” beetles used by Lopez et al. [9]. The following comparisons are only for the old and unmated treatments listed in Table 2 of the Lopez et al. [9] study. Our data on total distances flown by control females resembles Lopez et al. [9], but our beetles from the quarantine colony were slightly larger and heavier. In contrast, the males appeared to fly greater distances than the old, unmated males in the Lopez et al. [9] study. The beetles on our flight mills had fewer flight bouts than in the Lopez et al. [9] study, but it seems that the duration of flight bouts lasted longer. Javal et al. [10] also used laboratory-reared ALB adults on flight mills but found a similar trend to our findings in which the majority of flight bouts were shorter distances (<1.5 km), and they also observed a female that flew >14 km in its lifetime. Our results corroborated some of these findings from these ALB flight mill studies and showed that >15% of ALB adults were capable of dispersing >6 km after maturation and feeding, at least based on flight mills. However, we must stress that data from flight mills are not entirely realistic as flight mills supported the beetle’s weight, but also prevented feeding during the trials [9,29]. We also hypothesize that with no possibility of standing on a substrate, beetles were flying more than they might in nature. In addition, our laboratory-reared ALB colony could be more capable of flying great distances, in part due to their larger body size compared to the field-collected beetles used in the Lopez et al. [9] study. Thus, these data provide information about the effect of fungal infection on flight behavior but should not be used as a precise indication of flight behavior under field conditions (i.e., propensity to fly or flight distances).

Although ALB adults tend to stay on natal trees or on nearby trees during their lifetime [6], dispersal models based on records of infested trees in the Worcester, MA quarantine have suggested that some adults could disperse as far as 8 km [8]. The rare individuals that disperse as far as 8 km in their lifetime could escape quarantine zones and start new infestations [8]. In general, studies on the flight behavior of non-migrating insects, including ALB in the present study, have reported a right-skewed distribution in which most of the tested insects flew short distances but a small percentage flew greater distances [11, 29]. Flight mills may exaggerate the flight performance of ALB adults while they are tethered and not challenged by environmental stimuli, but they are crucial tools to quantify dispersal behavior and measure the impacts of experimental treatments [9,10,29]. Computerized flight mills allowed us to measure the impacts of a lethal pathogen on ALB flight capacity during the days preceding mortality. Although the main goal of using F52 in quarantine zones is killing ALB adults, reduced flight performance prior to ALB death would be another benefit gained from using this biological control agent.

## Supporting information

S1 Fig Survival time of F52-treated beetles in previous bioassays. Bar graph for the survival times of F52-treated *A*. *glabripennis* adults in different lab experiments that used the same inoculation method, but with a higher dose of 1.0 x 10^8^ conidia mL^-1^. The F52-treated beetles for the flight mill trials were exposed to 1.0 x 10^7^ conidia mL^-1^. Survival time is on the y-axis. Bar shading represents beetle sex; white bars for males (*n* = 46) and grey bars for females (*n* = 46).
(TIF)

S2 Fig Survival time of F52-treated beetles used for flight mill trials. Scatter plot for F52-treated *A*. *glabripennis* adults with survival time on the y-axis and total distance flown on the x-axis. Data is combined for flight mill trials from all time points (3, 7, and 10 DAT). The R^2^ statistic and *P*-value are provided next to the regression lines to show whether slope is significantly non-zero.
(TIF)

S1 Table Bioassays and flight mill trials. Dates of the 16 bioassays and subsequent flight mill trials at different time points using a random subsample of beetles for each bioassay date.
(DOCX)

S1 File Macros and sample flight mill data (online). Microsoft Excel macros for summarizing the raw data and listing the durations of individual flight bouts. LabView logs timestamps for each rotation of the flight mill arm for eight different flight mills. The raw data is exported from the LabView software as a text file. The text file is opened with Microsoft Excel before running the macros.
“Remove blanks” macro eliminates blank cells from the raw data and sorts the timestamps into new columns for each flight mill.
“Summarize bouts” macro identifies different flight bout events for each flight mill and calculates the duration of the flight bouts in seconds.
An excel spreadsheet from flight mill trials conducted on January 17, 2019 –January 18, 2019 includes raw data and a summary of the flight bouts for each flight mill.
Online link: https://figshare.com/articles/Excel_macros_and_data_example/9235142.
(ZIP)
