# Supplementary figures and images for: Impacts of Metarhizium brunneum F52 infection on the flight performance of Asian longhorned beetles, Anoplophora glabripennis
Source: PLoS One. 2019 Sep 6;14(9):e0221997. doi: 10.1371/journal.pone.0221997 (PMC6730868; doi:10.1371/journal.pone.0221997)

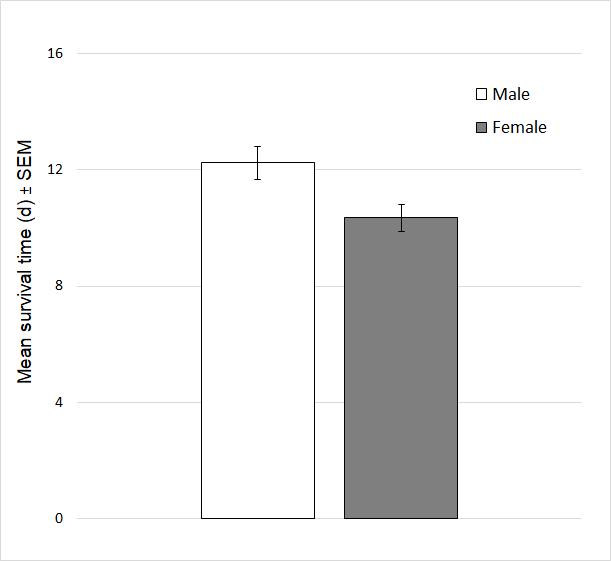

Supplement: S1 Fig — Bar graph for the survival times of F52-treated A. glabripennis adults in different lab experiments that used the same inoculation method, but with a higher dose of 1.0 x 10^8^ conidia mL-1. The F52-treated beetles for the flight mill trials were exposed to 1.0 x 10^7^ conidia mL-1. Survival time is on the y-axis. Bar shading represents beetle sex; white bars for males (n = 46) and grey bars for females (n = 46). (TIF) [file pone.0221997.s001.tif]

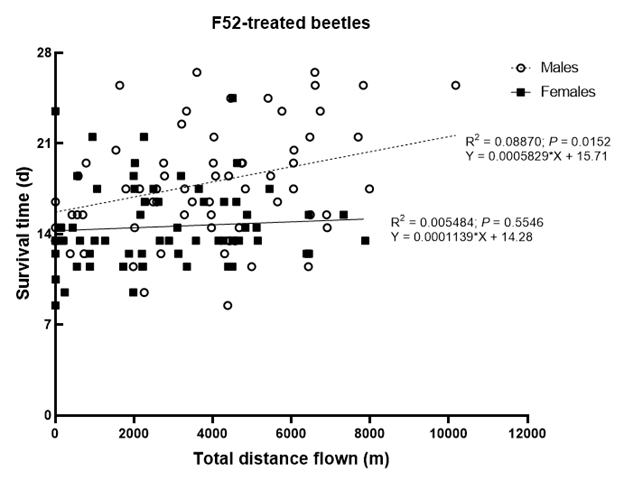

Supplement: S2 Fig — Scatter plot for F52-treated A. glabripennis adults with survival time on the y-axis and total distance flown on the x-axis. Data is combined for flight mill trials from all time points (3, 7, and 10 DAT). The R2 statistic and P-value are provided next to the regression lines to show whether slope is significantly non-zero. (TIF) [file pone.0221997.s002.tif]
