# Supplementary material for: Impacts of Metarhizium brunneum F52 infection on the flight performance of Asian longhorned beetles, Anoplophora glabripennis
Source: PLoS One. 2019 Sep 6;14(9):e0221997. doi: 10.1371/journal.pone.0221997 (PMC6730868; doi:10.1371/journal.pone.0221997)
Supplement: S1 Table — Dates of the 16 bioassays and subsequent flight mill trials at different time points using a random subsample of beetles for each bioassay date. (DOCX) [file pone.0221997.s003.docx]

**Supplementary Table 1.** Dates of the 16 bioassays and subsequent flight mill trials at different time points using a random subsample of beetles.

| **Date of bioassay** | **3 DAT* flight trials** | **7 DAT flight trials** | **10 DAT flight trials** |
| --- | --- | --- | --- |
| 6/4/2018 | 6/7/2018 | 6/11/2018 | Not done |
| 6/11/2018 | 6/14/2018 | 6/18/2018 | Not done |
| 6/25/2018 | 6/28/2018 | 7/2/2018 | 7/5/2018 |
| 7/9/2018 | 7/12/2018 | 7/16/2018 | 7/19/2018 |
| 7/23/2018 | 7/26/2018 | 7/30/2018 | 8/2/2018 |
| 8/6/2018 | 8/9/2018 | 8/13/2018 | 8/16/2018 |
| 8/20/2018 | 8/23/2018 | 8/27/2018 | 8/30/2018 |
| 9/3/2018 | 9/6/2018 | 9/10/2018 | 9/13/2018 |
| 9/17/2018 | 9/20/2018 | 9/24/2018 | 9/27/2018 |
| 9/24/2018 | Not done | 10/1/2018 | 10/4/2018 |
| 10/8/2018 | Not done | 10/15/2018 | 10/18/2018 |
| 10/15/2018 | Not done | 10/22/2018 | 10/25/2018 |
| 10/22/2018 | Not done | 10/29/2018 | 11/1/2018 |
| 11/19/2018 | Not done | 11/26/2018 | 11/29/2018 |
| 1/7/2019 | Not done | 1/14/2018 | 1/17/2018 |
| 1/14/2019 | Not done | Not done | 1/24/2019 |
| **16 bioassays (total)** | **9 (total)** | **15 (total)** | **14 (total)** |
|  | **38 flight mill trials (total)** | | |

*DAT: days after treatment (bioassay)
